# Involvement of Nitric Oxide in a Rat Model of Carrageenin-Induced Pleurisy

**DOI:** 10.1155/2010/682879

**Published:** 2010-06-02

**Authors:** Masahiro Iwata, Shigeyuki Suzuki, Yuji Asai, Takayuki Inoue, Kenji Takagi

**Affiliations:** ^1^Department of Rehabilitation, Faculty of Health Sciences, Nihon Fukushi University, 26-2 Higashihaemi-cho, Handa 475-0012, Japan; ^2^Department of Physical Therapy, Nagoya University School of Health Sciences, 1-1-20 Daikominami, Higashi-ku, Nagoya 461-8673, Japan; ^3^Department of Rehabilitation, Nagoya University Hospital, 1-1-20 Daikominami, Higashi-ku, Nagoya 461-8673, Japan; ^4^Program in Radiological and Medical Laboratory Sciences, Nagoya University Graduate School of Medicine, 1-1-20 Daikominami, Higashi-ku, Nagoya 461-8673, Japan

## Abstract

Some evidence indicates that nitric oxide (NO) contributes to inflammation, while other evidence supports the opposite conclusion. To clarify the role of NO in inflammation, we studied carrageenin-induced pleurisy in rats treated with an NO donor (NOC-18), a substrate for NO formation (L-arginine), and/or an NO synthase inhibitor (S-(2-aminoethyl) isothiourea or N^G^-nitro-L-arginine). We assessed inflammatory cell migration, nitrite/nitrate values, lipid peroxidation and pro-inflammatory mediators. NOC-18 and L-arginine reduced the migration of inflammatory cells and edema, lowered oxidative stress, and normalized antioxidant enzyme activities. NO synthase inhibitors increased the exudate formation and inflammatory cell number, contributed to oxidative stress, induced an oxidant/antioxidant imbalance by maintaining high O_2_^−^, and enhanced the production of pro-inflammatory mediators. L-arginine and NOC-18 reversed the proinflammatory effects of NO synthase inhibitors, perhaps by reducing the expression of adhesion molecules on endothelial cells. Thus, our results indicate that NO is involved in blunting—not enhancing—the inflammatory response.

## 1. Introduction

Inflammation is a protective process essential for the preservation of the integrity of organisms in the event of physical, chemical, or infectious damage. Acute inflammation, which is characterized by pain, heat, redness, and swelling, involves a complex series of events including vasodilatation, increased permeability, fluid exudation, and migration of leukocytes to the site of inflammation [1].

At the site of an inflammatory reaction, the injured vascular endothelial cells and the emigrated leukocytes synthesize and release an intricate cascade of inflammatory mediators including nitric oxide (NO). NO is an oxidant synthesized *de novo* in cells from L-arginine by isoforms of NO synthase (NOS), including neuronal NOS (nNOS or NOS1), inducible NOS (iNOS or NOS2), and endothelial NOS (eNOS or NOS3) [2]. Generally, nNOS and eNOS are constitutively expressed, whereas iNOS is induced in response to inflammatory-like stimuli and is capable of sustained production of high levels of NO that predominate during inflammation [2, 3]. Although the cytostatic/cytocidal activity of NO is part of the host defense mechanism, the excessive or inappropriate production of NO can damage tissue, possibly through the formation of peroxynitrite (ONOO^−^), a potent oxidizing and nitrating agent, via the coupling of NO with superoxide anion (O_2_^−^) [4, 5]. N^G^-monomethyl-L-arginine (L-NMMA), which is an arginine analogue that competitively inhibits the constitutive isoforms of NOS (cNOS: eNOS and nNOS) and iNOS, significantly reduced NO levels and concomitantly reduced synovial inflammation and tissue damage in streptococcal cell wall (SCW)-induced arthritis [6] and adjuvant arthritis [7] in rodents. Furthermore, NO-mediated production of ONOO^−^ in humans occurs in oxidative processes associated with atherogenesis [8] and acute lung injury [9]. Although this evidence suggests that NO plays a pro-inflammatory role, some reports contradict this finding. For example, the stimulation of endogenous NO production or exogenous administration of NO-donating compounds often blunts the ultimate expression of tissue injury at both the molecular and functional levels in cerebrovascular [10], hepatic [11], splanchnic [12], renal [13], pulmonary [14], myocardial [15], inflammatory, and ischemia/reperfusion injury models. Interestingly, this NO-dependent protective effect occurs in processes with increased rates of O_2_^−^ production and for which oxidant injury plays an etiological role [10, 11].

Carrageenin (Cg)-induced pleurisy in rats is a well-characterized experimental model of acute inflammation that permits the quantification and correlation of both exudate and cellular migration with changes in other inflammatory parameters [16]. In this model, polymorphonuclear leukocytes are the predominant cell type for 12 hours after Cg injection; then, they are replaced by migrating mononuclear cells that differentiate into macrophages and dominate the reaction until resolution at 48 hours [17, 18]. Thus, this model is suitable for studying the relationships among migrating cells, exudates, NO, pro-inflammatory cytokines, and chemokines.

The maintenance of leukocyte recruitment during inflammation requires intercellular communication between infiltrating leukocytes and the endothelium. These events are mediated by early response cytokines such as interleukin-1*β* (IL-1*β*) and tumor necrosis factor-*α* (TNF-*α*), cell-surface adhesion molecules, and chemotactic molecules such as chemokines [19].

Chemokines are a family of structurally related glycoproteins with potent leukocyte activation and/or chemotactic activity. Most chemokines are produced in response to inflammatory stimuli, such as the early-response cytokines, TNF-*α*, IL-1*β*, C5a, leukotriene B4, and interferons [20]. Monocyte chemoattractant protein-1 (MCP-1) is a chemokine that attracts monocytes and neutrophils both in vitro and in vivo, and it appears to be a key part of leukocyte emigration by promoting the transition from leukocyte rolling to adhesion on the endothelial surface [21].

In the present study, we analyzed the involvement of NO on inflammatory cell migration, nitrite/nitrate values, lipid peroxidation, and pro-inflammatory mediators (TNF-*α*, IL-1*β*, MCP-1 and O_2_^−^) in Cg-induced pleurisy in rats by administering NOC-18 (a donor of NO) [22], L-arginine (a substrate for NO formation), S-(2-aminoethyl) isothiourea (AE-ITU: an inhibitor of iNOS) [23], and N^G^-nitro-L-arginine (L-NNA: an inhibitor of cNOS and iNOS) [24] in combination with Cg injection.

## 2. Materials and Methods

### 2.1. Reagents

S-(2-aminoethyl) isothiourea (AE-ITU), N^G^-nitro-L-arginine (L-NNA), and 1-Hydroxy-2-oxo-3,3-bis(2-aminoethyl)- 1-triazene (NOC-18) were purchased from Dojindo (Kumamoto, Japan). Unless otherwise noted, all other chemicals used were of the purest grade and obtained from Sigma Chemical (St. Louis, MO, USA).

### 2.2. Animals

Male Wistar rats (Japan SLC, Shizuoka, Japan) weighing 150 to 170 g were used in this study. Food and water were available ad libitum. The light cycle was automatically controlled (12-hour light, 12-hour dark), and the room temperature was maintained at 23 ± 1°C. For 4 days prior to the experiments, animals were housed under these conditions to allow for acclimatization. All experiments were performed according to the Guidelines for Animal Experimentation of Nagoya University.

### 2.3. Induction of Cg-Induced Pleurisy and Measurement of Inflammatory Parameters

Animals were slightly anesthetized with diethyl ether; subsequently, 0.15 mL of physiological saline solution or 1% (w/v) *λ*-Cg (Sigma Chemical, St. Louis, MO, USA) suspended in physiological saline was injected into the right pleural cavity. After 2, 6, 12, 24, 36, 54, and 72 hours, animals were sacrificed via exsanguination from the abdominal aorta under deep ether-anesthesia. Pleural exudate from each animal was harvested by washing the pleural cavity with 1 mL of 3.15% (w/v) sodium citrate in physiological saline as an anticoagulant. Exudates contaminated with blood were rejected. Exudate volumes were measured, and the volume injected into the pleural cavity (1 mL) was subtracted from the total volume recovered. Exudate samples were then centrifuged at 800 × g for 10 minutes, after which cell pellets were resuspended in saline. To estimate the number of cells in each sample, cells were stained with trypan blue and then counted by using an optical microscope with a Burker counting chamber. In some experiments, differential cell counts were determined in smears by May-Grunwald staining.

### 2.4. Treatments

In the first set of experiments, rats (6 to 8 rats per group) received an intrapleural Cg injection immediately after intrapleural administration of vehicle (saline), 10 mg/kg of AE-ITU, 1 mg/kg of L-NNA, 10 mg/kg of NOC-18, or 30 mg/kg of L-arginine. The doses of AE-ITU [25], L-NNA [26], NOC-18 [27], and L-arginine [27] were based on previous reports from the literature with modification of the administration route.Five additional groups of rats(5 rats per group) were treated with 10 mg/kg of AE-ITU, 1 mg/kg of L-NNA, 10 mg/kg of NOC-18, or 30 mg/kg of L-arginine, but they did not receive the Cg injection. After 6 hours, the rats were sacrificed, and pleural exudates were collected and processed as described above.

In the second set of experiments, rats (6 rats per group) treated with AE-ITU (10 mg/kg) or L-NNA (1 mg/kg) immediately before Cg injection also received intrapleural injections of NOC-18 (10 mg/kg) or L-arginine (30 mg/kg). NOC-18 and L-arginine treatments were performed simultaneously with NOS inhibitor injection. Animals were sacrificed 6 hours after the induction of pleurisy, and pleural exudates were collected and processed. In each set of experiments, the control group received only vehicle(s) via the appropriate route of administration.

### 2.5. Nitrite/Nitrate Assay

The amount of nitrites and nitrates, which are indicators of NO synthesis, in the supernatant of centrifuged exudate was measured with a colorimetric commercial kit (Nitrite/Nitrate Assay Kit-C II; Dojindo), according to the manufacturer's instructions.

### 2.6. Malondialdehyde Assay

We used the thiobarbituric acid reactive substances (TBARS) method as an index of lipid peroxidation for analyzing malondialdehyde (MDA) products during an acid-heating reaction, as previously described by Draper and coworkers [28]. Briefly, the supernatant of 200 *μ*L of centrifuged exudates was mixed with 1 mL of 10% trichloroacetic acid and 1 mL of 0.67% thiobarbituric acid; the samples were then heated in a boiling water bath for 30 minutes. TBARS levels were determined by absorbance at 532 nm and expressed as malondialdehyde equivalents.

### 2.7. Assay for TNF-*α*, IL-1*β*, and MCP-1

TNF-*α*, IL-1*β* and MCP-1 were measured in the supernatant of centrifuged exudates by enzyme-immunoassay kits (BioSource International, Camarillo, CA, USA), according to the manufacturer's instructions.

### 2.8. Total Antioxidant Status Assay

The total antioxidant status (TAOS) is an indication of O_2_^−^ and other oxidant species. The TAOS of the supernatant of centrifuged exudate was determined as previously described [29]. Briefly, the reaction mixture consisted of (final concentrations): 20 *μ*L 2,2′-azino-bis(3-ethylbenzthiazoline-6-sulfonic acid) (ABTS; 2 mM), 10 *μ*L horseradish peroxidase (30 mU/mL), 20 *μ*L H_2_O_2_ (0.1 mM), 40 *μ*L phosphate-buffered saline (PBS; in mM: 137 NaCl, 8.10 Na_2_HPO_4_, 2.68 KCl, 1.47 KH_2_PO_4_, pH 7.40), and 10 *μ*L of sample in a total volume of 100 *μ*L per well of 96-well plates. The reaction, which was initiated upon the addition of H_2_O_2_, was conducted at 37°C. The increase in absorbance at 405 nm, a reflection of 2,2′-azino-bis(3-ethylbenzthiazoline-6-sulfonic acid) radical cation (ABTS^*+*^) accumulation, was measured by using a microplate reader (Molecular Devices, Crawley, UK).

### 2.9. Statistical Analysis

All data are expressed as the mean ± standard error of the mean (S.E.M.). The statistical analysis was conducted by using ANOVA followed by the Bonferroni *t*-test. Differences between groups were considered statistically significant when *P* < .05.

## 3. Results

### 3.1. Time Course of Exudate Formation and Cellular Infiltration

**Figure 1:**
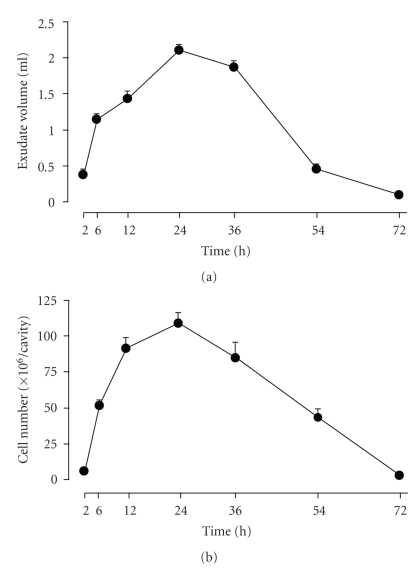
Time course of rat Cg pleurisy. Exudate volume (a) and leukocyte infiltration (b) were evaluated at different time points after Cg injection. The values are expressed as mean ± S.E.M. of 6 to 8 rats.

The pleural cavity of control rats that did not receive Cg or drugs contained no exudates and 2.9 ± 0.5 × 10^6^ cells per rat (*n* = 5), and these cells were predominantly mononuclear (>95%). Injection of 0.15 mL of 1% Cg into the pleural cavity of rats caused an inflammatory response characterized by exudate formation and cell migration (Figure 1). The exudate volume and the number of leukocytes in the pleural cavity increased with time, reaching a maximum at 24 hours (2.11 ± 0.08 mL/rat and 108.6 ± 7.5 × 10^6^ cells/rat, *n* = 8) and decreasing at 54 hours (0.46 ± 0.07 mL/rat and 43.3 ± 6.1 × 10^6^ cells/rat, *n* = 7). Both levels returned to the background levels by 72 hours. Differential cell counts of leukocytes that migrated into the pleural cavity showed that polymorphonuclear leukocytes dominated the early phase (2 hours) of the reaction (89% polymorphonuclear leukocytes and 11% mononuclear leukocytes) and were then replaced at 54 hours by mononuclear leukocytes (26% polymorphonuclear leukocytes and 74% mononuclear leukocytes).

### 3.2. Time Course of Nitrite/Nitrate Production in the Exudate of Cg-Induced Rat Pleurisy

**Figure 2:**
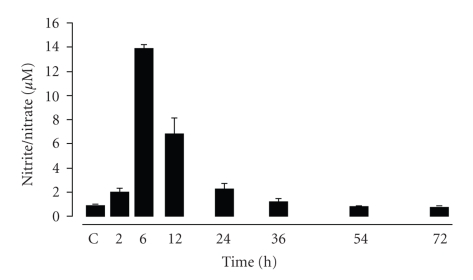
Time course of nitrite/nitrate levels. The nitrite/nitrate assay was performed in rat pleural exudates collected from control rats (C) or Cg-treated rats at 2, 6, 12, 24, 36, 54, and 72 hours after pleural injection. The values are expressed as mean ± S.E.M. of 6 to 8 rats.

We measured the levels of nitrite/nitrate in the pleural exudate of rats at different time points after the induction of pleurisy. As shown in Figure 2, nitrite/nitrate was detectable in the pleural exudate at 2 hours and peaked at 6 hours after Cg injection before gradually decreasing.

### 3.3. Effects of AE-ITU, L-NNA, NOC-18, or L-Arginine on Cg-Induced Rat Pleurisy

**Figure 3:**
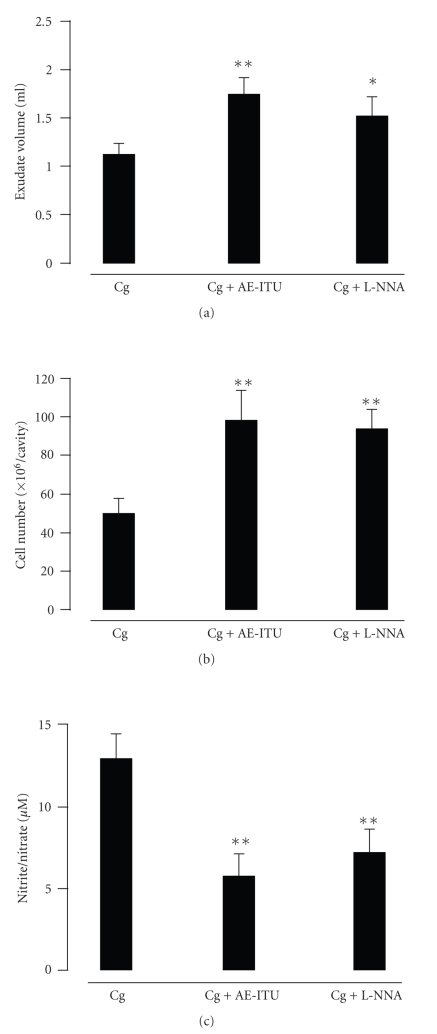
Effects of AE-ITU and L-NNA on rat Cg pleurisy and nitrite/nitrate levels. AE-ITU or L-NNA was injected into the pleural cavity immediately before Cg injection. The exudate volume (a), cell number (b), and nitrite/nitrate levels (c) in the pleural exudates were determined 6 hours after Cg injection. Data are expressed as mean ± S.E.M. of 6 to 8 rats. *P < .05, **P < .01 as compared to the Cg group.

To determine the contribution of NO to the inflammatory response, we measured the effects of AE-ITU (selective iNOS inhibitor), L-NNA (non-selective NOS inhibitor), NOC-18 (a donor of NO), or L-arginine (a substrate for NO formation) administered directly into the pleural cavity immediately before Cg injection. The exudate formation and inflammatory cell number were significantly increased with the addition of AE-ITU (both *P* < .01) (Figures 3(a) and 3(b)) or L-NNA (*P* < .05 and *P* < .01, resp.). Both the Cg + AE-ITU and Cg + L-NNA groups showed a marked reduction in the levels of nitrite/nitrate as compared to the Cg group (both *P* < .01) (Figure 3(c)). No statistical differences were observed between the Cg + AE-ITU and Cg + L-NNA groups.

**Figure 4:**
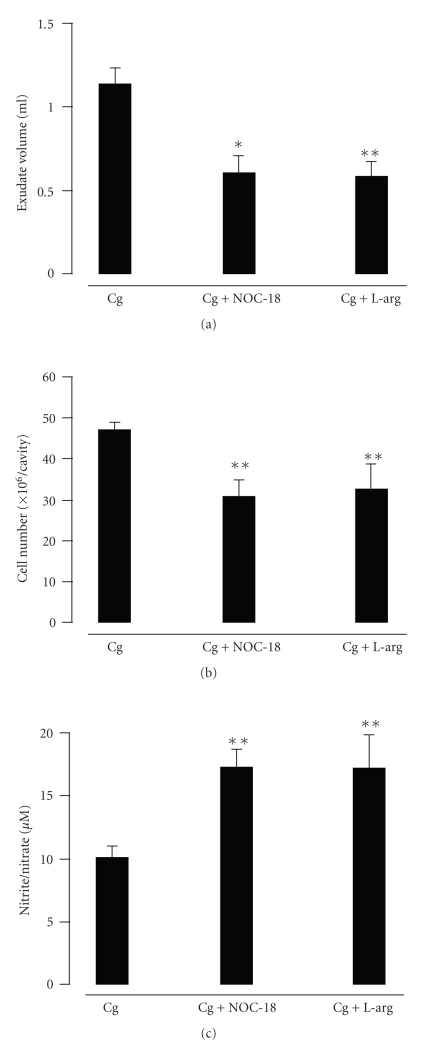
Effects of NOC-18 and L-arginine on rat Cg pleurisy and nitrite/nitrate levels. NOC18 or L-arginine was injected into the pleural cavity immediately before Cg injection. The exudate volume (a), cell number (b), and nitrite/nitrate levels (c) in the pleural exudates were determined 6 hours after Cg injection. Data are expressed as mean ± S.E.M. of 6 to 8 rats. *P < .05, **P < .01 as compared to the Cg group.

In a parallel experiment, the exudate volume and the number of inflammatory cells were significantly reduced by the addition of NOC-18 (*P* < .05 and *P* < .01) (Figure 4(a)) or L-arg (both *P* < .01) (Figure 4(b)). Both the Cg + NOC-18 and Cg + L-arg groups had markedly increased levels of nitrite/nitrate as compared to the Cg group (both *P* < .01) (Figure 4(c)).

### 3.4. Effects of NOC-18, or L-Arginine on the Influence of AE-ITU or L-NNA on Cg-Induced Rat Pleurisy

**Figure 5:**
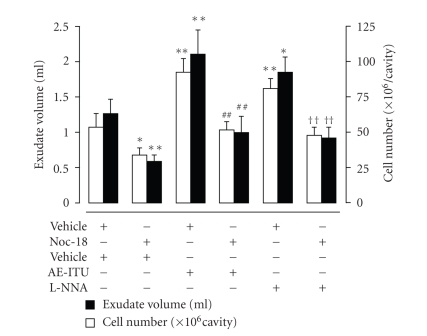
Effects of NOC-18 on the influence of AE-ITU and L-NNA on exudate volume (empty columns) and cell infiltration (filled columns) 6 hours after Cg challenge. Each column represents the mean ± S.E.M. of 6 rats. *P < .05, **P < .01 as compared to the vehicle plus vehicle; ^##^ *P* < .01 as compared to the vehicle plus AE-ITU; ^††^ *P* < .01 as compared to the vehicle plus L-NNA.

Next, we determined if the changes in the NO level were responsible for the exacerbation of inflammation at 6 hours by AE-ITU and L-NNA. When NOC-18 was injected in rats simultaneously treated with AE-ITU or L-NNA, the exacerbating influences of these NOS inhibitors were completed reversed (Figure 5). Similar results were obtained with L-arginine (data not shown).

### 3.5. Effects of AE-ITU, L-NNA, NOC-18 or L-Arginine on Pleural Inflammation in the Absence of Cg

To show that AE-ITU, L-NNA, NOC-18 or L-arginine did not affect inflammation simply as a result of direct irritation, these compounds or vehicle were injected into the pleural cavity in the absence of Cg. None of these compounds caused exudate formation or an increase in the cellular influx (data not shown).

### 3.6. Effect of AE-ITU, L-NNA, NOC-18, or L-Arginine on Malondialdehyde Content in Pleural Exudates

**Figure 6:**
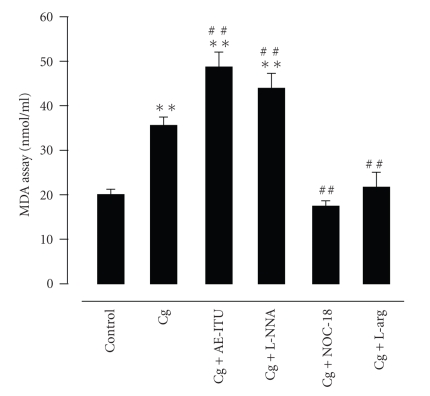
Effect of AE-ITU, L-NNA, NOC-18, and L-arginine on malondialdehyde (MDA) content in the pleural exudates 6 hours after Cg challenge. Data are expressed as mean ± S.E.M. of 6 to 8 rats. **P < .01 as compared to control group; ^##^ *P* < .01 as compared to Cg group.

The MDA levels in the Cg (*P* < .01), Cg + AE-ITU (*P* < .01) and Cg + L-NNA groups (*P* < .01) were significantly increased as compared to the control group (Figure 6). MDA levels in the Cg + AE-ITU and Cg + L-NNA groups were increased as compared to that of the Cg group (both *P* < .01). On the other hand, the Cg + NOC-18 and Cg + L-arg groups had a decreased MDA level as compared to the Cg group (both *P* < .01). MDA levels in the Cg + NOC-18 and Cg + L-arg groups were similar to that of the control group.

### 3.7. Effect of AE-ITU, L-NNA, NOC-18, or L-Arginine on the Release of TNF-*α*, IL-1*β*, and MCP-1

**Figure 7:**
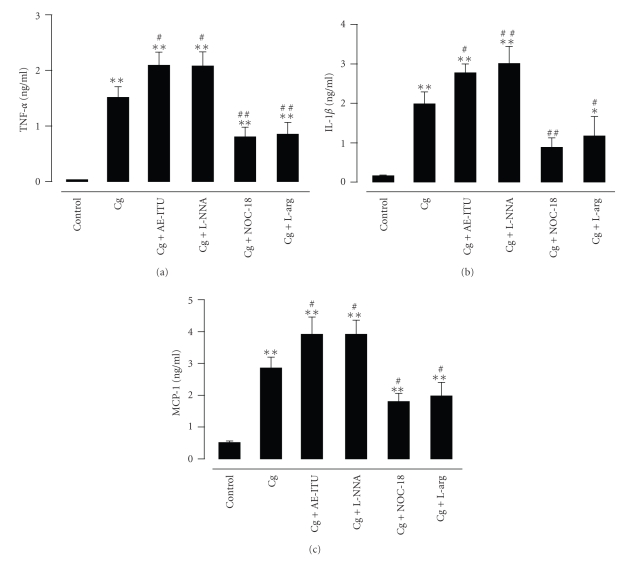
Effects of AE-ITU, L-NNA, NOC-18 and L-arginine on the release of cytokines. Tumor necrosis factor-*α* (TNF-*α*) (a), interleukin-1*β* (IL-1*β*) (b) and monocyte chemoattractant protein-1 (MCP-1) (c) levels in the pleural exudates 6 hours after Cg challenge. Data are expressed as mean ± S.E.M. from 6 to 8 rats. *P < .05, **P < .01 as compared to control group; ^#^ *P* < .05, ^##^ *P* < .01 as compared to Cg group.

We measured the levels of TNF-*α* (Figure 7(a)), IL-1*β* (Figure 7(b)), and MCP-1 (Figure 7(c)) in the pleural exudates. The Cg group had a higher level of TNF-*α* as compared to the control group (*P* < .01). The Cg + AE-ITU and Cg + L-NNA groups had the highest levels of TNF-*α* in comparison to the Cg group (both *P* < .05) and the control group (both *P* < .01). The levels of TNF-*α* in the Cg + NOC-18 and Cg + L-arg groups were lower than that of the Cg group (both *P* < .01) but higher than that of the control group (both *P* < .01). The Cg (*P* < .01), Cg + AE-ITU (*P* < .01) and Cg + L-NNA groups (*P* < .01) had increased IL-1*β* levels as compared to the control group, while the Cg + NOC-18 (*P* < .01) and Cg + L-arg groups (*P* < .05) had reduced IL-1*β* levels as compared to the Cg group. However, in the Cg + L-arg group, the level of IL-1*β* was higher compared to the control group (*P* < .05), while there was no difference between the IL-1*β* levels of the Cg + NOC-18 and control groups. Like the TNF-*α* and IL-1*β* levels, the MCP-1 levels in the Cg + AE-ITU and Cg + L-NNA groups were increased as compared to the Cg group (both *P* < .05). The MCP-1 levels of the Cg + NOC-18 and Cg + L-arg groups were significantly lower than that of the Cg group (both *P* < .05) and significantly higher than that of the control group (both *P* < .01).

### 3.8. Effect of AE-ITU, L-NNA, NOC-18 or L-Arginine on TAOS Activity in Pleural Exudates

**Figure 8:**
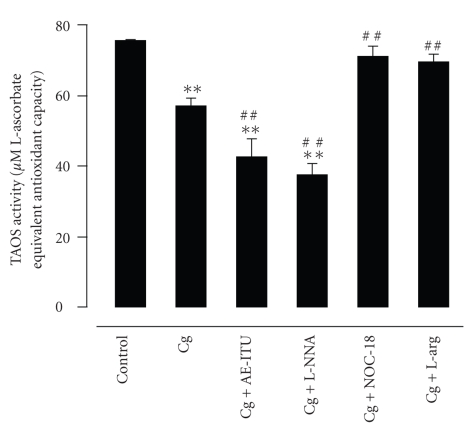
Effect of AE-ITU, L-NNA, NOC-18, and L-arginine on total antioxidant status (TAOS) activity in pleural exudates 6 hours after Cg challenge. Data are expressed as mean ± S.E.M. from 6 to 8 rats. **P < .01 as compared to the control group; ^##^ *P* < .01 as compared to the Cg group.

The Cg group had a lower level of TAOS activity as compared to the control group (*P* < .01) (Figure 8). The Cg + AE-ITU and Cg + L-NNA groups had the lowest level of TAOS activity in comparison to the Cg group (both *P* < .01) and the control group (both *P* < .01). In contrast, the TAOS activity levels in the Cg + NOC-18 and Cg + L-arg groups were increased in comparison to the Cg group (both *P* < .01) and similar to that of the control group.

## 4. Discussion

Nitric oxide is a signaling molecule responsible for diverse physiological and pathophysiological processes. Until now, the prevailing hypothesis about NO is that it contributes to toxicant-induced pleural inflammation and injury [30, 31]. The present study suggests a different role for NO in the rat pleural inflammatory response induced by Cg. We found that the administration of an NO donor (NOC-18) or NO substrate (L-arginine) reduced the migration of inflammatory cells and edema formation, lowered oxidative stress, and normalized antioxidant enzyme activities. The action of NO here was different from its actions elicited by other pleura and/or lung inflammatory stimuli such as lipopolysaccharide (LPS), des-Arg9-bradykinin, or substance P [32–34]. Although several studies have described NO as a mediator of the inflammatory response—for example, by stimulating production of inflammatory cytokines and peroxynitrite (ONOO^−^) [4, 5]—beneficial effects of NO during inflammatory insult have also been reported [10–15, 25, 27].

We observed a reduction in the number of inflammatory cells within the pleural cavity in the Cg + NOC-18 and Cg + L-arg groups as compared to the Cg group. The specific action of NO on inflammatory cells is unknown, but we suggest that NOC-18 and L-arginine may reduce the expression of endothelial cell adhesion molecules and consequently reduce the inflammatory cell migration into the pleural space. Furthermore, NO was reported to be an important molecule for blocking the endothelium-leukocyte interaction [35, 36]. Apoptosis of inflammatory cells during the inflammatory process by Cg injection through NO activation of death domains is another hypothesis to explain the reduced number of inflammatory cells within the pleural cavity in the Cg + NOC-18 and Cg + L-arg groups, due to the involvement of NO in apoptosis [36, 37]. By contrast, L-NNA, an inhibitor of cNOS and iNOS, or AE-ITU, a selective iNOS inhibitor, resulted in an increased level of leukocytes as compared to the Cg group. Therefore, although Cg itself increased nitrite/nitrate formation, decreasing the nitrite/nitrate formation with L-NNA or AE-ITU actually exacerbated the inflammatory response in terms of leukocyte levels and exudate volume. The effects of the NOS inhibitors on leukocyte migration appear to be a consequence of the inhibition of the L-arginine/NO pathway, because the enhancement of leukocyte migration was reversed by cotreatment with L-arginine and NOC-18. These data help to explain the positive effects of an NO donor or substrate in decreasing the inflammatory response by lowering cell recruitment into the inflammatory site. However, it is possible that the contribution of NO to the development of the inflammatory reaction depends on the intensity of tissue injury and integrity of the structures involved in the process. Future efforts also will undoubtedly focus on attempting to decipher the differences in the time course of pharmacological assays, doses, and the observation time intervals.

The fact that the IC_50_ of L-NNA for the inhibition of eNOS is around 10 times lower than that for iNOS inhibition [24] suggests that, at low doses, L-NNA is inhibiting predominantly the eNOS isoform. This, together with the demonstration that AE-ITU, a selective inhibitor of iNOS [23], enhanced the leukocyte migration, suggests that during the inflammatory process, NO, which modulates the leukocyte migration, is synthesized by either eNOS or iNOS. There is evidence in the literature that during the inflammatory process the expression of eNOS or its activity is enhanced [38] and iNOS is induced [39]. Additionally, other authors have demonstrated that NO downmodulates the leukocyte migration during the inflammatory process and that it is released by both NOS isoforms. Animals treated with selective iNOS inhibitors (aminoguanidine, 1400W, L-NIL and AE-ITU) or a selective eNOS inhibitor (L-NIO) present enhanced leukocyte migration to inflammatory sites [25, 27, 40, 41]. Furthermore, the NO donors, SIN-1, spermine-NO, sodium nitroprusside, and NOC-18, inhibit both the adhesion and leukocyte migration induced by LPS, IL-1, Cg, or ischemia/reperfusion [27, 42–45]. On the other hand, there are some studies that contradict these reports and our own findings. Animals treated with NOS inhibitors (L-NAME, L-NIL, L-NMMA, or aminoguanidine) or iNOS knockout mice present a reduction in the leukocyte migration induced by staphylococcal enterotoxin B (SEB), zymosan, SWC, or Cg [40, 46–48]. It has been suggested that these apparently conflicting data could be a consequence of vasoconstriction, leading to reduced local blood flow, due to the use of high doses and/or systemic administration of the drugs [25, 27]. However, decreased blood flow in the microcirculation cannot completely explain the reduction in leukocyte migration induced by zymosan and Cg in iNOS knockout mice [46, 47], which do not experience changes in blood pressure after the inflammatory stimuli injection. We do not have an explanation for these differences; however, in our study, rats treated with L-NNA or AE-ITU and injected with Cg presented a massive leukocyte migration. It is important to point out that rats treated with the same dose of L-NNA did not present a significant increase in blood pressure [26]. 

Lipid peroxidation is an important marker of oxidative stress in pleuritis. Cg-induced pleurisy and lipid peroxidation (as analyzed by MDA) have been previously associated [28]. We found that MDA levels in Cg-injected animals were increased by NOS inhibitors. Treatment with NOC-18 or L-arginine reduced the amount of MDA. This result is similar to that of Machová and coworkers [49], who showed that NO treatment inhibited lipid peroxidation in vitro and protected against cellular damage and cytotoxicity. NO has also been described as a scavenger of other far more toxic radicals and, therefore, an enhancer of defense mechanisms [50]. Consequently, the increased levels of lipid peroxidation were observed only in the Cg + L-NNA and Cg + AE-ITU groups but not in the Cg + NOC-18 and Cg + L-arg groups.

There is substantial evidence that the pro-inflammatory cytokines TNF-*α* and IL-1*β* propagate the extension of a local or systemic inflammatory process [51, 52]. We confirm here that the inflammatory process induced by Cg injection into the pleural cavity leads to substantial increases in the levels of TNF-*α* and IL-1*β* in the exudate. Interestingly, the inhibition of NO synthesis by L-NNA or AE-ITU further increased the levels of these two pro-inflammatory cytokines in the exudate, whereas NOC-18 or L-arginine did not cause a further increase. In support of this observation, others have shown that inhibition of NO synthesis by L-NAME in rats subjected to hepatic ischemia/reperfusion enhanced the expression of TNF-*α* and IL-1*β* mRNA in the ischemic lobes of liver and increased plasma levels of TNF-*α* and IL-1*β* [53]. Previous experiments have also shown that the NO donor SNAP dose dependently reduced the amounts of TNF-*α* and IL-1*β* produced by activated macrophages [54].

MCP-1 is a monocyte chemotactic factor that exerts potent and specific chemoattractant activity on both monocytes and neutrophils [21]. Monocytes and, to a lesser extent, granulocytes secrete MCP-1 in response to cytokines, viruses, bacterial endotoxins, and mitogens [55]. In vitro studies have shown that NO donors (NONOate compounds) inhibit the expression and production of MCP-1 in cytokine-activated human endothelial cells [56]. In addition, inhibition of endogenous NO synthesis by L-NNA increased endothelial MCP-1 mRNA expression and resulted in a marked increase in monocyte chemotactic activity [57], suggesting that NO modulates MCP-1 expression and activity in vitro. In agreement with these studies, we found that MCP-1 production in pleural exudates is downregulated by NOC-18 or L-arginine and upregulated by NOS inhibitors. Collectively, these results suggest that a compensatory increase in NO production at inflammatory sites critically regulates the severity of the inflammatory response by moderating the levels of such potently pro-inflammatory mediators as TNF-*α*, IL-1*β*, and MCP-1. Additionally, the lower levels of TNF-*α*, IL-1*β*, and MCP-1 in the Cg + NOC-18 and Cg + L-arg groups confirm the efficacy of treatment with an NO donor or substrate, leading us to hypothesize that NO is involved in blunting the inflammatory response.

We measured TAOS activity as an indirect indication of the formation of O_2_^−^ and other oxidant species. This assay is based on the principle that oxidants formed during inflammation react with antioxidants such as glutathione, ascorbic acid, and *α*-tocopherol [29] and reduce the antioxidant capacity of inflammatory exudates [58]. This index of O_2_^−^ and other oxidant species was increased in the Cg + NOC-18 and Cg + L-arg groups in comparison with the Cg group, and these values were generally similar to those of the control group. Thus, the Cg + NOC-18 and Cg + L-arg groups appeared to downregulate O_2_^−^ generation to some degree. In contrast, the TAOS activities of both the Cg + L-NNA and Cg + AE-ITU groups were lower than those of the Cg and control groups, suggesting that O_2_^−^ generation was enhanced in the absence of NO. O_2_^−^ is produced by polymorphonuclear leukocytes and macrophages from the enzyme activity of NADPH oxidase and xanthine oxidase at inflammatory sites. Both enzyme systems contain a heme prosthetic group with which NO can react to inhibit O_2_^−^ release [59]. Therefore, inhibiting NO removes the brakes on O_2_^−^ production. In support of this notion, others have shown that NO generation reduces O_2_^−^ levels, while its inhibition increases O_2_^−^ production both in vitro and in vivo [60, 61]. As O_2_^−^ has been associated with tissue damage and loss of function during inflammatory episodes [62], it is conceivable that one of the contributors to an enhanced inflammatory response, consequent to NOS inhibition, is O_2_^−^ generation. Indeed, elevated levels of O_2_^−^ increase TNF-*α* release from macrophages [63] and neutrophils and neutrophil activation through a Toll-like receptor 4-dependent mechanism [64]. Increased levels of O_2_^−^ also activate NF-*κ*B [65], which may account for the increased production of IL-1*β*. Although no direct effects of O_2_^−^ on the upregulation of IL-1*β* have been demonstrated, an O_2_^−^ dismutase mimetic, M40403, significantly reduced NF-*κ*B DNA binding [66] and IL-1*β* production in inflamed lungs [67]. Moreover, in endothelial cells, adenovirus-mediated expression of O_2_^−^ dismutase suppressed TNF-*α*-induced MCP-1 mRNA expression and MCP-1 protein secretion [68]. Therefore, a disturbance in the balance between NO and O_2_^−^ production may lead to an increase in pro-inflammatory mediators and provide a possible mechanism for the exacerbation of inflammation observed in the present study.

In summary, we showed that NO supplementation by NOC-18 or L-arginine reduced Cg-induced pleurisy and oxidative stress and regulated TNF-*α*, IL1-*β*, MCP-1, and O_2_^−^ levels at the inflammatory site. The NOS inhibitors exacerbated the inflammatory status in the pleural cavity of Cg-injected rats, contributed to oxidative stress, and induced a major imbalance between oxidants and antioxidants by maintaining high O_2_^−^ levels, favoring the oxidant side. Supplementation with NO may be a promising treatment for inflammatory diseases that involve oxidative stress, but further investigations are needed to confirm this hypothesis.
